# 

**Published:** 1916-04-08

**Authors:** 


### Gallant R.A.M.C. Officers.

The list of rewards for gallantry and devotion to duty published on March 31 contains the names of four officers of the Royal Army Medical Corps who receive the Military Cross in recognition of their heroism. Captain Bernard Grellier, attached 10th Royal Welsh Fusiliers, was conspicuous for his bravery when tending the wounded under heavy shell fire; he also helped to dig out wounded men who were buried. Temporary Captain L. D. Saunders, attached 10th Notts and Derby Regiment, tended the wounded under heavy fire, and finally evacuated them all with great skill and care; his clothing was torn by fragments of shell, and he was at work for forty-eight hours without a rest. This is not the first time Captain Saunders has displayed great coolness and devotion to duty. Of Temporary Captain H. V. White, attached 7th Lincolnshire Regiment, it is recorded that he stuck to his work for thirty-six hours, although his dressing-station was repeatedly hit by shells and he himself was severely knocked about several times. Temporary Lieutenant J. A. Harper, 52nd Field Ambulance, behaved with conspicuous gallantry when leading stretcher-bearers during operations; it is recorded that on one occasion, when three of his bearers were wounded, he went alone, under heavy shell fire, to the aid post. The President of the French Republic has bestowed the decoration "Croix de Guerre" on two Territorial officers of the Royal Army Medical Corps—namely, Lieutenant-Colonel James Mill and Captain Eric Dalrymple Gairdner; and two officers of the Canadian Army Medical Corps are recipients of the like distinction—namely, Captain J. A. Cullum and Captain G. H. R. Gibson. This coveted honour has been granted in recognition of distinguished service during the campaign.

### Centenary of the Royal Waterloo Hospital.

The Royal Waterloo Hospital for Children and Women is one of the few hospitals which, by reason of the special character of their work, have been unable to participate in the treatment of wounded soldiers, and therefore cannot base their appeals for support on the martial sentiment of the times. It is interesting to note, however, that a large proportion of the patients are children of sailors and soldiers. The hospital was founded in 1816 as the "Royal Universal Dispensary for Children," which was then situated at St. Andrew's Hill, Doctors' Commons, its sole object being that "of affording prompt medical aid to the children of the necessitous poor, from the period of their birth to the age of twelve years, in all parts of the metropolis and its vicinity." This year, therefore, the institution reaches its centenary, and it is hoped to celebrate the occasion by raising £50,000 for H.R.H. the Duchess of Albany's Centenary Reserve Fund. The appeal, which in normal circumstances would have been begun eighteen months ago, has been delayed by the war, but on April 27 next, the birthday of this institution, an impetus will be given to the appeal by a meeting at the Mansion House. Part of the amount that will be raised will be used to defray the cost of a centenary wing which is to replace the insanitary and dilapidated buildings now containing the nurses' home and isolation wards. Contributions are to be sent to Mrs. Harkness, at the Royal Waterloo Hospital, Waterloo Road, S.E., and all donors of £5 and upwards will receive a letter of thanks from H.R.H. the Duchess of Albany.

### HOSPITAL RESULTS IN 1915.

### More Early Reports of the Voluntary Hospitals.

St. Andrew's Hospital, Dollis Hill, N.W.—This year's report has as a frontispiece a striking portrait of H.M. the Queen of the Belgians, who is Patroness of the hospital. The institution, founded in 1911, is now firmly established, and is doing most useful work. Two classes of patients are received : first, those of limited means, among whom are a number of French and Belgian refugees of superior social standing, who have received medical treatment and the services of nurses free of charge; and, second, many British and Belgian wounded soldiers, for whom there are now sixty beds. Of £5,027 total ordinary income, £3,416 is under the heading of "Patients' Payments."

Chesterfield and North Derbyshire Hospital.—Up to December 31, 1915, 120 British and 20 Belgian soldiers had been received in the wards, but owing to the heavy civilian waiting list it is arranged that no further soldier patients shall be admitted for the present. The income for the year was slightly higher than in 1914 and exceeded the expenditure by about £250. The out-patient total has fallen from 5,540 in 1910 to 3,737 last year.

Birmingham and Midland Hospital for Women.—The annual report, presented at the meeting of the governors on March 28, stated that owing to the demand for the admission of in-patients and the extent to which the services of the honorary medical staff had been requisitioned for military work, it had been found necessary to curtail the work of the out-patient department, where there were 2,246 attendances fewer than in the preceding year. The resignation of the matron, Miss Richmond, after eighteen years' service, was reported; a testimonial fund amounting to over £1,000 had been invested on her behalf in War Loan. The income of the hospital fell by £420 and the expenditure by £150. There was a deficit on the year's working of £2,324.

The Royal Devon and Exeter Hospital.—As a frontispiece to the annual report there is a charming photograph of the Bowring Ward for Children. The number of patients awaiting admission throughout the year was exceptionally high. The number of out-patients is being reduced by referring minor cases to the panel doctor. The ordinary income was £1,256 less than in 1914. £1,913 was earned by the private nursing staff. There was a deficit on the accounts of £2,865 and legacies amounting to £2,103 had to be used for current expenses.

The Infants' Hospital, Westminster.—Of the 337 cases treated to a conclusion in 1915, 138 died and 164 made good recoveries. The accounts show a deficit of £500 on the year's working, and the hospital is £1,256 in debt. 272,943 articles were washed in the hospital at a cost of £93. There are regular courses of lectures on infant feeding and management.

### GIFTS AND BEQUESTS.

Miss Gertrude Langton, formerly of Liverpool, left £300 to the Infirmary for Children, Myrtle Street, Liverpool.

The Baroness Biddulph bequeathed £300 to the Hereford Cottage Hospital and £50 to the Hereford County Nursing Association.

From the trustees of the late Colonel J. K. Holdsworth the Whitby Cottage Hospital has received a gift of £750 to endow a bed for local sea fishermen.

The Queen has sent a donation to the Italian Hospital in London, in recognition of the treatment of English and Belgian soldiers at that institution.

By the will of Lord Armitstead, the Royal Infirmary, Dundee, is to receive £10,000, the Sidlaw Sanatorium £1,000, and the Sick Poor Nursing Society £500.

Miss Elizabeth Nicholson, of Sheffield, who died on February 23, bequeathed £100 each to the Jessop Hospital for Women, the Royal Hospital, and the Royal Infirmary, Sheffield.

The Royal National Hospital for Consumption, Ventnor, has received a further gift of £1,000 War Loan Stock from Sir Ernest Cassel, to name a bed in memory of his daughter, Mrs. Ashley.

Three thousand pounde for the infirmary and £750 for the convalescent home have been received by the Edinburgh Royal Infirmary from the estate of Mr. John Croall, of Edinburgh. Miss Marian Sutherland has also bequeathed £2,000 to the infirmary.

Mr. Edwin Tildesley, of West Hampstead, left £1,000 each to the Royal Hospital for Incurables and St. Mary's Hospital, Paddington.

Manchester Royal Infirmary has received £950 from the Hospital Saturday Fund and £500 from Mr. Frederick Jackson (in memory of his late wife).

To perpetuate the memory of the late Dr. Mary Murdoch, of Hull, a fund has been opened permanently to endow a cot at the Hull Children's Hospital.

Ashton District Infirmary will receive at least £1,000 under the will of the late Mr. Thomas Taylor, of Marton, for the endowment of a cot in the children's wards.

Under the will of the late Mrs. William Robinson, of Blackpool, in memory of her husband, provision is made for the endowment of a bed at the Blackpool Hospital.

The Bluecoat Hospital, Wavertree, and the Infirmary for Children, Myrtle Street, Liverpool, each benefit to the extent of £300 under the will of Miss Gertrude Langton.

A sum of over £6,000 should be available for division between the Royal Infirmary, Bradford, and the Harrogate Infirmary, the two residuary legatees under the will of the late Mrs. Emily Ann Widdop.

Miss Myra Watson, of Porchester Terrace, who died on February 14, left £1,000 each to the Middlesex Hospital, the Samaritan Free Hospital, St. George's Hospital, and the School for the Blind, Swiss Cottage; £500 to the University of Cambridge for the Study of Physiology; and £500 to the St. Dunstan's Hostel for Blinded Soldiers and Sailors.

### PASSING EVENTS AND TOPICS.

### Handsome Gift to the Red Cross Fund.

A contribution of £10,000 has been received by the Scottish Red Cross branch from the coalmasters of Scotland.

### The Arrival of the "Sikkim."

The hospital ship *Sikkim* has arrived at Basra. She is a fast, comfortable ship, double-decked, and equipped with electric light and fans and an operating theatre, and accommodates 144 cots.

### Operations on Hungarian Soldiers.

The Hungarian Minister of Home Defence has issued an order to the effect that when fitness for service depends entirely on the performing of an operation, the military surgeon has the right to act on his own responsibility, without the consent of the patient.

### Manchester Police Ambulance.

At Manchester a special police ambulance corps has been formed with a view to service in the city should casualties occur during a raid. There are six fully equipped ambulance and dressing stations, at each of which there will be on emergency an officer in charge, two ambulance officers, and fifteen to twenty men, including one cyclist orderly.

### Motor Ambulances for the French Army.

In response to an invitation which had been sent to him by some of the members of Lloyd's who desire to record their sympathy with the soldiers of France by presenting a convoy of motor ambulances for the use of the French Army, Lord Beresford, as chairman of the British Ambulance Committee, visited Lloyd's last week and made an appeal on behalf of the fund.

### Improvised Defence against Asphyxiating Gases.

In view of the statement that bombs dropped during the recent Zeppelin raid contained asphyxiating gases, it is worth noting that the French military authorities advise that if appropriate masks are not available a serviceable device is to plug the nose with a damp handkerchief on which soap has been rubbed. The principle of the defence against asphyxiating fumes is the use of a humid alkaline barrier.

### The French Red Cross.

From August 2, 1914, the first day of mobilisation, to January 1, 1916, the *Société Française de Secours aux Blessés Militaires*, one of the three Societies which make up the French Red Cross, has administered 796 hospitals containing 67,081 beds. It is now organising at Salonika a hospital of 500 beds. The trained nurses of the Society number 15,000, of whom about 3,000 are serving in the military hospitals. According to the Paris correspondent of the official organ of the American Medical Association the Society's expenditure has amounted to over thirty-two million francs.

### Guardians Stand Firm.

By twenty votes to eight the Stockport Board of Guardians at its recent meeting decided to inform the Local Government Board that, having regard to all the special circumstances of the case and the careful consideration that had been given to the matter, the Guardians were still of the opinion that the appointment of Dr. J. Howie Smith (assistant medical officer) as medical officer was in the best interests of the Union, and they respectfully requested the Local Government Board to sanction the appointment. The Local Government Board had urged the desirability of a temporary appointment during the war.

### University College Hospital.

Sir Ernest Hatch, who presided at the recent annual meeting of this hospital, said that the civil population had in no way suffered by the admission of soldiers, as the beds had been increased from 305 to 447. Two thousand wounded soldiers had been received as in-patients, and over 15,000 as out-patients. Sir Ernest, in alluding to the high price of some drugs, said it seemed inconceivable to him that a plant like belladonna could not be cultivated on a sufficiently large scale in England to supply our requirements of sulphate of atropine. The financial position of the hospital continues satisfactory.

### East London Hospital for Children.

Mr. H. Machin presided at the annual court of the governors of the East London Hospital for Children, and while congratulating the meeting that things were no worse, said that the run of ill-fortune reported in previous years had not entirely stopped, as the indebtedness to the bankers had further increased by £200, and the amount now owing was £4,000. But considering the times the hospital was fortunate that the income had come so near the expenditure. The board have resolved to give a year's trial to a scheme whereby beds at the hospital and convalescent home are placed at the disposal of the London County Council for tuberculous patients. The convalescent home during 1915 had been closed to patients and had been lent for six months for billeting troops. It is a remarkable thing that an institution having such a strong claim upon the sympathy of the public should be in such an unpromising financial condition.

### King's College Hospital.

Lord Hambleden, chairman of the Committee of Management, and Mr. L. A. Martin, chairman of the General Appeal Committee, of King's College Hospital, write: "The hospital now erected at Denmark Hill is absolutely the latest and best equipped in the kingdom, but there is, unfortunately, a debt of something like £65,000 still to be paid off. At present the hospital is very largely occupied by wounded soldiers, but an annual sum of £11,000 has to be found for the current expenses over and above the War Office receipts and the ordinary income of the hospital. When the soldiers leave and the committee use the whole of the beds for civilians, the position will be extremely precarious, as the only revenue that can be depended upon is about £18,000, and an annual deficiency of about £30,000 may be expected." The removal of the hospital was carried out with the warm approbation of King Edward's Hospital Fund for London, which subscribed handsomely to the Removal Fund and contribute annually to the maintenance of the institution. The appeal is therefore endorsed by the highest guarantee that substantial financial support will be well placed. Gifts may be sent to the Hon. Treasurer, Mr. H. W. Twining, at 97 Cannon Street, E.C.

H.M. the Queen will open the new Chelsea Hospital for Women on Tuesday, July 11 next. The plans and a full description of this hospital were published in our issue of July 10, 1915.

### Personalia.

Dr. K. K. Robinson, who died at Dover on March 31, had been medical officer of health for Dover and East Kent for forty-three years.

Captain S. S. Greaves, of the 2nd West Riding Field Ambulance, R.A.M.C., is reported wounded. Prior to the war he was on the medical staff of Leeds General Infirmary.

Sir Arthur Lawley, who has served for over a year as Commissioner in France of the British Red Cross Society, has found it necessary to resign that post, in order to take up important work in London. Lord Donoughmore has been appointed to succeed him as Commissioner.

Dr. J. H. Whitaker, M.D., B.Ch., D.A.O. (R.U.I.), D.P.H. (Camb.), has been appointed by the Metropolitan Asylums Board medical superintendent of the Eastern Hospital, in succession to Dr. Goodall, who has been transferred to the North-Western Hospital. Dr. Whitaker has been in the service of the Board for nearly 18½ years, and has been a senior medical officer for close on 15½ years. He has acted as medical superintendent during annual leave periods, and also during Dr. Goodall's absences in France for two periods of three months each. Amongst the applicants applying for the position was Dr. Cameron, at present medical superintendent of the River Hospitals and Ambulance Service, but the Hospitals Committee was of opinion that he would be best serving the interests of the Board by remaining in his present position.

---

### The Book Market.

LIST OF NEW BOOKS RECEIVED FROM THE FOLLOWING PUBLISHERS:—

Constable and Co., Ltd.: "The Medical Register, 1916." 10s. 6d. "The Dentist's Register, 1916." 3s. 4d.

"Medical Homes for Private Patients, 1916." Eleventh year. Price 6d. net.

J. B. Lippincott Company: "A Text-book of Physics and Chemistry for Nurses." By A. R. Bliss, jun., Ph.G., A.M., M.D., and A. H. Olive, A.B., A.M., Ph.Ch. Price 6s. net. "Surgical and Gynæcological Nursing." By E. M. Parker, M.D., F.A.C.S., and S. D. Breckinridge, M.D., F.A.C.S. Price 10s. 6d. net.

J. Maclehose and Sons, Glasgow: "Bernhardi and Creation: A New Theory of Evolution." By Sir James Crichton-Browne, M.D., D.Sc., LL.D., F.R.S. Price 1s. net.

### Institutional and Public Reports.

In addition to those specially noticed annual reports have been received from the following: Department of Agriculture of the Province of Alberta, 1914; and the Medical Board of Health for Sheffield, 1914.

---

### The League of Roses.

At the annual meeting of the League of Roses—an Islington institution, whose record of work on behalf of the Great Northern Central Hospital continues with increasing success—it was stated that thirteen hundred collecting-boxes had been out during the year and £200 more than in 1914 had been collected. During last year £700 had been given to the hospital by the League.

RATES OF SUBSCRIPTION: Twelve months, 6/6; Six months, 4/-; Three months, 2/-, post free to any address in the United Kingdom. Abroad, Twelve months, 8/8; Six months, 5/-; Three months, 2/6, post free.—Address The Subscription Dept., "THE HOSPITAL," The Hospital Building, 28/29 Southampton Street, Strand, London, W.C.

